# Assessing cellular efficacy of bromodomain inhibitors using fluorescence recovery after photobleaching

**DOI:** 10.1186/1756-8935-7-14

**Published:** 2014-07-13

**Authors:** Martin Philpott, Catherine M Rogers, Clarence Yapp, Chris Wells, Jean-Philippe Lambert, Claire Strain-Damerell, Nicola A Burgess-Brown, Anne-Claude Gingras, Stefan Knapp, Susanne Müller

**Affiliations:** 1 Structural Genomics Consortium, Nuffield Department of Clinical Medicine, University of Oxford, Old Road Campus Research Building, Roosevelt Drive, Oxford OX3 7DQ, UK; 2 Target Discovery Institute, Nuffield Department of Clinical Medicine, University of Oxford, Roosevelt Drive, Oxford OX3 7FZ, UK; 3 Lunenfeld-Tanenbaum Research Institute, Mount Sinai Hospital, 600 University Avenue, Toronto, ON M5G 1X5, Canada; 4 Department of Molecular Genetics, University of Toronto, Toronto, ON M5S 1A8, Canada; 5 Current address: Botnar Research Centre, Nuffield Department of Orthopaedics, Rheumatology and Musculoskeletal Sciences, University of Oxford, Windmill Road, Oxford OX3 7LD, UK; 6 Current address: Nanotether Discovery Sciences Ltd, Museum Avenue, Cardiff University, Cardiff CF10 3AX, UK

**Keywords:** Bromodomain, Cell-based assay, Confocal microscopy, Epigenetics, Fluorescence recovery after photobleaching, Histone acetylation, Small-molecule inhibitor

## Abstract

**Background:**

Acetylation of lysine residues in histone tails plays an important role in the regulation of gene transcription. Bromdomains are the readers of acetylated histone marks, and, consequently, bromodomain-containing proteins have a variety of chromatin-related functions. Moreover, they are increasingly being recognised as important mediators of a wide range of diseases. The first potent and selective bromodomain inhibitors are beginning to be described, but the diverse or unknown functions of bromodomain-containing proteins present challenges to systematically demonstrating cellular efficacy and selectivity for these inhibitors. Here we assess the viability of fluorescence recovery after photobleaching (FRAP) assays as a target agnostic method for the direct visualisation of an on-target effect of bromodomain inhibitors in living cells.

**Results:**

Mutation of a conserved asparagine crucial for binding to acetylated lysines in the bromodomains of BRD3, BRD4 and TRIM24 all resulted in reduction of FRAP recovery times, indicating loss of or significantly reduced binding to acetylated chromatin, as did the addition of known inhibitors. Significant differences between wild type and bromodomain mutants for ATAD2, BAZ2A, BRD1, BRD7, GCN5L2, SMARCA2 and ZMYND11 required the addition of the histone deacetylase inhibitor suberoylanilide hydroxamic acid (SAHA) to amplify the binding contribution of the bromodomain. Under these conditions, known inhibitors decreased FRAP recovery times back to mutant control levels. Mutation of the bromodomain did not alter FRAP recovery times for full-length CREBBP, even in the presence of SAHA, indicating that other domains are primarily responsible for anchoring CREBBP to chromatin. However, FRAP assays with multimerised CREBBP bromodomains resulted in a good assay to assess the efficacy of bromodomain inhibitors to this target. The bromodomain and extraterminal protein inhibitor PFI-1 was inactive against other bromodomain targets, demonstrating the specificity of the method.

**Conclusions:**

Viable FRAP assays were established for 11 representative bromodomain-containing proteins that broadly cover the bromodomain phylogenetic tree. Addition of SAHA can overcome weak binding to chromatin, and the use of tandem bromodomain constructs can eliminate masking effects of other chromatin binding domains. Together, these results demonstrate that FRAP assays offer a potentially pan-bromodomain method for generating cell-based assays, allowing the testing of compounds with respect to cell permeability, on-target efficacy and selectivity.

## Background

Posttranslational modification of histones represents an important mechanism for the epigenetic regulation of gene expression. ϵ-N-acetyl lysine marks on histones can be ‘written’ and ‘erased’ by histone acetyltransferases (HATs) and histone deacetylases (HDACs), respectively, and are ‘read’ by bromodomains. Sixty-one unique bromodomains have been identified in forty-two diverse human proteins, which function as transcriptional regulators, chromatin remodelling factors, splicing factors, scaffold proteins and signal transducers, or have additional epigenetic functions such as methyltransferase or HAT activity [1]. In many cases however, the function of a specific bromodomain-containing protein remains unknown. In addition, bromodomain-containing proteins have been implicated in a wide range of diseases. Overexpression of numerous family members has been reported in a variety of cancers [2-6], and translocations of *BRD3* or *BRD4* with *NUT* and *CREBBP* with *MLL*, *MOZ* or *MORF* have been observed in NUT midline carcinoma [7] and acute myeloid and lymphoblastic leukaemia [8], respectively. Bromodomain-containing proteins have also been implicated in proinflammatory processes as well as in a number of neurological diseases [2,3]. The involvement of bromodomain-containing proteins in such a wide range of diseases makes them attractive therapeutic targets, and, as a result, a number of bromodomain inhibitors have been entered into clinical trials [9-13].

Although bromodomains exhibit considerable sequence diversity, they share a conserved fold that comprises a left-handed bundle of four α-helices, which form the acetyl-lysine binding pocket. A highly conserved asparagine residue in this binding pocket is typically responsible for anchoring the acetyl-lysine side chain via hydrogen bonding, but can in some cases be replaced by other amino acids, including threonine or tyrosine [1]. This deep structurally conserved and largely hydrophobic cavity provides a viable target for the development of acetyl-lysine competitive inhibitors. We have previously described biochemical assays for the identification of small-molecule inhibitors of several diverse bromodomains [14]. A key step in the development of bromodomain inhibitors is the demonstration of cellular efficacy for the target of interest, which is complicated by the functional diversity, or even unknown function, of many bromodomain-containing proteins.

Fluorescence recovery after photobleaching (FRAP) has evolved into a powerful confocal microscopy technique in which a portion of a live cell bearing fluorescently labelled molecules is photobleached by a high-intensity laser pulse and the migration of labelled molecules back into the bleached area is monitored over time [15]. This technique can be applied to the analysis of bromodomain-binding to chromatin, where- the protein of interest is fused to a fluorescent protein, such as green fluorescent protein (GFP). After photobleaching, diffusion of unbleached protein back into the bleached region is retarded by protein binding to chromatin and chromatin-associated complexes and is therefore slower compared to a freely diffusible molecule. Thus, the time taken for recovery is related to protein affinity, and an inhibitor of protein binding would be expected to reduce recovery time [16].

Since bromodomains lack any catalytic activity that could otherwise be monitored and the common denominator in the function of most bromodomain-containing proteins is chromatin association, FRAP represents a target agnostic method for detecting bromodomain inhibition. Furthermore, direct visualisation of an on-target effect in the nucleus of live cells also offers the advantage of eliminating artefacts associated with *ex vivo* assays. Indeed, we have previously described displacement of BRD4 from chromatin by small-molecule inhibitors in a FRAP-based assay [17,18]. Here we investigate whether FRAP assays have the potential to be used broadly across the bromodomain family for establishing cellular efficacy of inhibitors.

## Results and discussion

**Figure 1:**
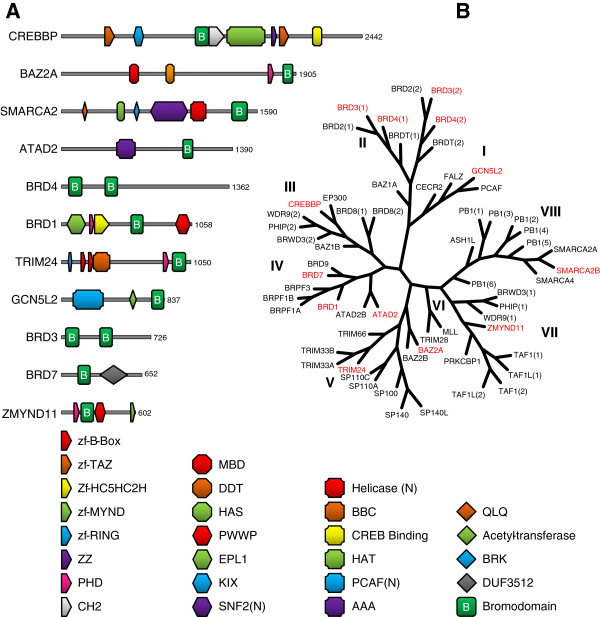
**Bromodomain phylogenetic tree and domain structure of representative family members. (A)** Domain organization of bromodomain-containing proteins for which fluorescence recovery after photobleaching (FRAP) assays are described herein. Protein length in amino acids is shown at the right of each protein. The identities of the different domains are given in the legend at the bottom. **(B)** The structure-based phylogenetic tree of the human bromodomain family according to Filippakopoulos *et al*. [1]. The different families are named by Roman numerals (I to VIII). Proteins for which FRAP assays are described herein are highlighted in red.

Bromodomain-containing proteins have been identified as attractive therapeutic targets [3]. In order to assess the on-target effect of developed inhibitors in the intact cell, we employed FRAP experiments for a variety of these targets broadly covering the branches of the bromodomain tree [1] (Figure 1B). We started with BRD4, for which we have previously demonstrated target engagement in the cell [17,18].

BRD4, which contains two bromodomains (Figure 1A), binds to acetylated histones and remains associated with chromatin throughout mitosis, providing a mechanism for epigenetic memory that maintains efficient postmitotic transcription of ‘bookmarked’ genes [19]. During interphase, BRD4 also plays a key role in the regulation of transcriptional elongation by recruiting the positive transcription elongation factor b, or P-TEFb, complex to promoters and thereby facilitating the phosphorylation and activation of RNA polymerase II [20]. Although these known functions provide possible downstream biological readouts for BRD4 bromodomain inhibition, FRAP assays allow the disruption of chromatin binding to be directly visualised in living cells.

**Figure 2:**
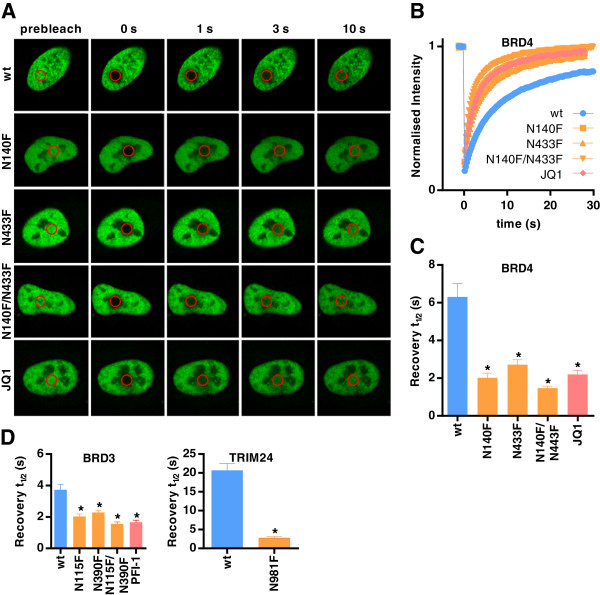
**Fluorescence recovery after photobleaching assays detect mutation of the bromodomains and inhibition by small molecules. (A)** Nuclei of U2OS cells transfected with plasmids encoding green fluorescent protein fused to wild-type BRD4, BRD4 bromodomain mutants or wild-type BRD4 treated with JQ1. The bleached area is indicated by a red circle. **(B)** Time dependence of fluorescence recovery in the bleached area for the BRD4 fluorescence recovery after photobleaching (FRAP) assay. Curves represent the means at each time point of at least ten cells in each group. Half-times of fluorescence recovery (t½) in FRAP assays for BRD4 **(C)** and BRD3 and TRIM24 **(D)**. Bars represent the mean t½ calculated from individual recovery curves of at least ten cells per group, and error bars depict the standard error of the mean. Where an inhibitor is used, concentration is 1 μM. wt, Wild type; N###F, Bromodomain mutants, indicating substitution made. *P < 0.05, significant difference from wild type.

Full-length, GFP-tagged wild-type and mutant BRD4 proteins localised exclusively to the nucleus but were excluded from the nucleoli (Figure 2A), as has been reported for endogenous BRD4 [21]. Photobleaching of a 13.6 μm^2^ area of the nucleus (approximately 6%) resulted in gradual recovery to >90% of initial intensities (Figure 2B), indicating that the majority of the GFP-tagged protein is mobile, with a half recovery time (t½) of 6.3 ± 0.7 seconds for the wild-type protein (Figure 2C). Mutation of one bromodomain (N140F, located in the first bromodomain of BRD4 or N443F in the second bromodomain of BRD4) or both bromodomains (N140F and N433F) at the conserved asparagine that forms a hydrogen bond with acetylated lysines of histone tails resulted in a significantly reduced t½ (*P* < 0.05). This implies that BRD4 is being anchored to chromatin, at least in part, via the bromodomains. Previous *in vitro* studies have demonstrated that the first bromodomain of BRD4 binds more tightly to some histone acetylation sites than the second domain [1], and this trend was also seen in FRAP, although significance was not reached by analysis of variance (ANOVA) multiple comparisons testing. The observed reduction in FRAP recovery times between the wild type and mutants demonstrates that a measurable assay window should exist for the displacement of wild-type BRD4 from chromatin by small-molecule inhibitors of the bromodomain. Indeed, this was seen for the bromodomain and extraterminal protein (BET) bromodomain inhibitor JQ1 [17], which reduced t½ to levels similar to the double-mutant when added 1 hour prior to FRAP. The short incubation time also argues strongly that the observed effects of JQ1 are due to direct displacement of BRD4 rather than to downstream consequences of inhibition of endogenous BRD4.

Similar results were also observed in FRAP for BRD3 [22,23], another member of the BET subfamily containing two bromodomains (Figure 2D). t½ was significantly reduced relative to the wild type for first bromodomain (N115F), second bromodomain (N390F) and double-bromodomain (N115F/N390F) mutants (*P* < 0.05). A second BET inhibitor based on a different chemotype, PFI-1 [18,24], reduced t½ to levels similar to that of the double-mutant, confirming in the FRAP assay the potency observed *in vitro* against both bromodomains.

TRIM24 has an N-terminal tripartite motif, consisting of a RING domain, two B-box domains and a coiled-coil region, that is characteristic of the TRIM family, in addition to the C-terminal PHD-bromodomain cassette (Figure 1A). TRIM24 acts as a transcriptional regulator, binding to chromatin and interacting with several nuclear receptors and coactivators [25]. A clear reduction in t½ was seen between wild-type TRIM24 (20.7 ± 1.8 seconds) and the N981F mutant (2.8 ± 0.3 seconds) (Figure 2D). Small-molecule inhibitors of the TRIM24 bromodomain are yet to be described, but the pronounced difference between the wild type and bromodomain mutant suggest that this FRAP assay would be sensitive to such compounds. Of note is the long recovery time of the wild-type TRIM24, despite the use of a small bleach area (2.5 μm^2^), which is consistent with previous *in vitro* findings where TRIM24 exhibited the greatest reported affinity between a bromodomain and an acetylated histone peptide [3,26]. This affinity appears to be conferred by the PHD and bromodomain acting as a single functional unit for the combinatorial recognition of unmodified H3K4 and H3K23ac within the same histone tail.

**Figure 3:**
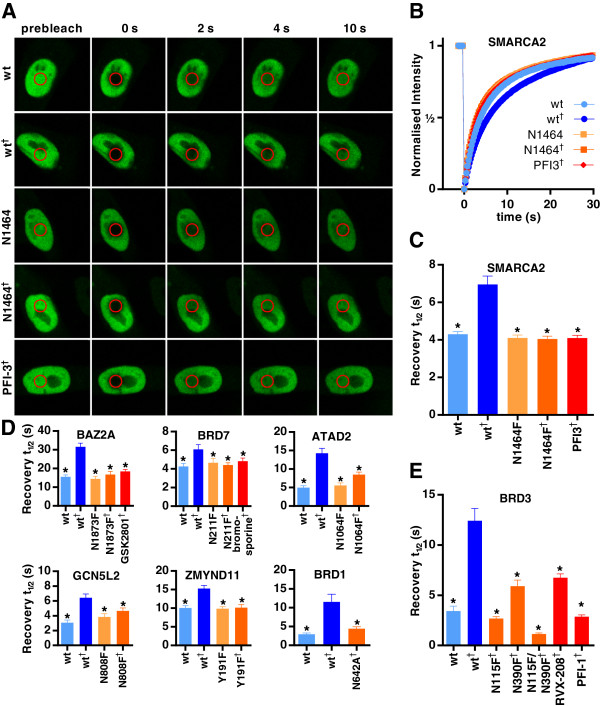
**Fluorescence recovery after photobleaching assays performed with the addition of suberoylanilide hydroxamic acid to establish a robust assay window. (A)** Nuclei of U2OS cells transfected with plasmids encoding green fluorescent protein chimerised to wild-type or mutant SMARCA2, with or without 2.5 μM suberoylanilide hydroxamic acid (SAHA) and the inhibitor PFI-3. The bleached area is indicated by a red circle. **(B)** Time dependence of fluorescence recovery in the bleached area for the SMARCA2 fluorescence recovery after photobleaching (FRAP) assay. Half-times of fluorescence recovery (t½) in the FRAP assays for SMARCA2 **(C)**, BAZ2A, BRD7, ATAD2, GCN5L2, ZMYND11 and BRD1 **(D)** and BRD3 **(E)**. Bars represent the mean t½ calculated from individual recovery curves of at least ten cells per group, and error bars depict the standard error of the mean. Where an inhibitor is used, concentration is 1 μM, except GSK2801, which is 5 μM. wt, Wild type; X####F, Bromodomain mutants, indicating substitution made. ^†^Addition of 2.5 μM SAHA. *P < 0.05, significant difference from wild type^†^.

SMARCA2 is a central component of the SWI/SNF chromatin remodelling complex and contains multiple chromatin binding domains [27] (Figure 1A). When FRAP experiments were performed with full-length SMARCA2, no significant difference in recovery times could be observed between the wild type and bromodomain mutants (see Figure 3C and Additional file 1: Figure S1), indicating that SMARCA2 chromatin binding was not being primarily driven through bromodomain interactions in unstimulated U2OS cells. However, preincubation with the HDAC inhibitor suberoylanilide hydroxamic acid (SAHA; 0.625 to 10 μM), which would be expected to increase the global levels of histone acetylation, resulted in a dose-dependent increase in t½ (see Additional file 1: Figure S1). This increase in FRAP recovery time was clearly being driven by interaction with the bromodomain, since the N1464F mutant did not respond to SAHA preincubation (*P* > 0.05). Although the greatest effect of SAHA preincubation was seen at higher concentrations, cytotoxicity was also pronounced above 2.5 μM (data not shown), reducing the number of viable cells available for FRAP analysis. Therefore, 2.5 μM SAHA was used in all subsequent experiments where the addition of SAHA was necessary to create an assay window.

**Figure 4:**
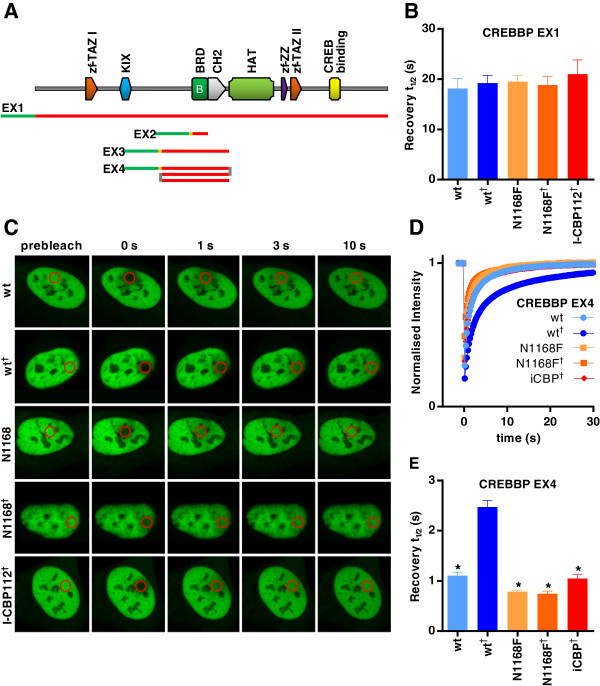
**Fluorescence recovery after photobleaching assay utilising a multimerised CREBBP bromodomain construct to establish a robust assay window. (A)** Domain organisation of CREBBP and representation of the regions of CREBBP incorporated into various green fluorescent protein (GFP) chimeric expression constructs. **(B)** Half-times of fluorescence recovery (t½) from fluorescence recovery after photobleaching (FRAP) assay using full-length CREBBP (EX1). **(C)** Nuclei of U2OS cells transfected with plasmids encoding GFP chimerised to wild-type (wt) or mutant multimerised CREBBP bromodomain (EX4), with or without 2.5 μM suberoylanilide hydroxamic acid (SAHA) and the inhibitor I-CBP112. The bleached area is indicated by a red circle. **(D)** Time dependence of fluorescence recovery in the bleached area of cells expressing wt or mutant EX4. **(E)** Half-times of fluorescence recovery (t½) of cells expressing wt or mutant EX4. Bars represent the mean t½ calculated from individual recovery curves of at least ten cells per group, and error bars depict the standard error of the mean. Where an inhibitor is used, concentration is 1 μM. N####F, Bromodomain mutants, indicating substitution made. ^†^Addition of 2.5 μM SAHA. *P < 0.05, significant difference from wt^†^.

Full-length, GFP-tagged wild-type and mutant SMARCA2 proteins were localised exclusively in the nucleus, but not in nucleoli (Figure 3A), consistent with reported subcellular localisation [28]. Photobleached areas recovered to >95% of their initial intensities, indicating that the SMARCA2 protein is highly mobile (Figure 4C). t½ in the wild-type control cells was 3.6 ± 0.1 seconds, which was increased to 5.6 ± 0.3 seconds by the addition of SAHA (*P* < 0.05). Mutation of the bromodomain (N1464F) resulted in recovery times indistinguishable from those of wild-type controls, irrespective of SAHA addition, and the increase in t½ seen in wild-type cells with the addition of SAHA was abolished by the SMARCA2 inhibitor PFI-3 [29] (*P* < 0.05), demonstrating that the assay is sensitive to small-molecule inhibitors.

Addition of SAHA was also necessary to establish viable assay windows for BAZ2A [30], BRD7 [31,32], ATAD2 [33], GCN5L2 [34] and ZMYND11 [35,36] (Figure 3D). For ZMYND11 FRAP, recovery times of bromodomain mutants differed little from those of wild-type controls and were unchanged by the addition of SAHA, indicating that the observed increase in wild type t½ in response to SAHA was mediated solely by the bromodomain. In the BAZ2A, BRD7, ATD2 and GCN5L2 FRAP assays, there was a consistent trend for the SAHA-treated mutant cells to show longer FRAP recovery times compared to the wild-type or mutant cells, although this difference was not always significant in ANOVA multiple comparisons testing. This could be due to SAHA-induced global chromatin acetylation resulting in a shift toward the euchromatin state, allowing greater opportunity for binding to chromatin through non-bromodomain-driven interactions. Despite this partial response of mutant cells to SAHA treatment, a clear assay window (*P* < 0.05) could be seen between the SAHA-treated wild-type and mutant cells, implying that these assays would be sensitive to small-molecule inhibitors. Indeed, where such inhibitors have been described (GSK2801 [37] for BAZ2A and bromosporine [38] for BRD7), reduced recovery times in the presence of SAHA back to SAHA-treated mutant control levels were observed (*P* < 0.05). Of note is the response of wild-type, but not mutant, ZMYND11 to SAHA, since ZMYND11 has an atypical bromodomain that does not contain the conserved asparagine present in all of the other bromodomains examined here, but rather a tyrosine, suggesting that FRAP assays may also be applicable to other atypical bromodomain-containing proteins.

When BRD1 [39] was first evaluated for FRAP under the same conditions as those in other assays, GFP-tagged BRD1 appeared to be localised exclusively to distinct nuclear speckles, without any diffuse distribution across the nucleus, consistent with reported immunohistological staining results [40]. Attempted FRAP assays with these cells produced almost no recovery after photobleaching (data not shown), indicating that BRD1 is almost entirely immobilised in these speckles within the time frame of the FRAP experiments. Speckling was observed with both wild-type and mutant BRD1 and was unaffected by the addition of SAHA, suggesting that this distribution is not being driven by the bromodomain. However, when microscope gain settings were increased to >850 to allow visualisation of cells expressing very low levels of GFP-BRD1, cells with a homogeneous distribution of tagged protein could be observed. Initial experiments with these cells showed an appreciable immobile fraction (approximately 20%), and no difference in t½ was observed between cells expressing wild-type or mutant BRD1 in the absence of SAHA (data not shown). However, the FRAP recovery time of wild-type cells, but not mutant cells, was significantly increased (*P* < 0.05) by the addition of SAHA (Figure 3D), indicating that a viable FRAP assay is possible if only cells with low levels of BRD1 expression are selected.

In the FRAP assays for BRD3 and BRD4, both of which contain two bromodomains, assays sensitive to small-molecule inhibitors were possible without the addition of SAHA. However, although there was a trend in both assays for mutation of the first bromodomain to produce a greater reduction in t½ than the second, which would be consistent with *in vitro* affinity studies [41,42], these differences failed to reach significance in ANOVA multiple comparisons testing. In an attempt to improve the sensitivity of these assays, a BRD3 FRAP assay was performed with the addition of SAHA, which resulted in a clear significant difference (*P* < 0.05) between the N115F and N390F mutants (Figure 3E). Furthermore, the assay was able to differentiate between the inhibitors RVX0208, which is reported to inhibit primarily the second domain of BRD3 [41,42] and produces a t½ almost identical to that of the N390F mutant, and PFI-1, which is active against both bromodomains and reduces t½ to a much greater extent. Use of these inhibitors at the higher dose of 5 μM ensures that differences are due to selective targeting of the bromodomains rather than to differences in potency.

CREBBP, and its paralog EP300, are involved in many physiological processes, including proliferation, differentiation and apoptosis [43]. Both act as transcriptional coactivators for a large number of transcription factors and exhibit chromatin-remodelling properties through relaxation of chromatin through intrinsic HAT activity [44]. FRAP experiments with full length CREBBP produced no discernable difference in recovery time between the wild type and the bromodomain mutants (Figure 4B). Furthermore, addition of SAHA did not increase the t½ of wild-type cells, and the system was unresponsive to inhibitors. The moderately long t½ of 14.9 ± 1.1 seconds for the wild-type control cells indicates that CREBBP is binding to chromatin, but the lack of effect of bromodomain mutation or either SAHA or inhibitor addition suggests that the CREBBP bromodomain is not a major driver of this interaction. Indeed, CREBBP possesses several other chromatin binding domains (Figure 1A), including two TAZ zinc finger domains, a KIX domain, a ZZ-type zinc finger domain and the CREB binding domain, all of which are involved in binding to other transcription factors [45-48], as well as the HAT domain that acetylates histone and nonhistone proteins [49]. Together, all of these chromatin interactions could be masking any contribution by the bromodomain.

To isolate chromatin–bromodomain interactions from other CREBBP binding domains, a number of expression constructs were made (Figure 4A) with either N- or C-terminal GFP fusions. However, transfection of constructs with C-terminal GFP was poorly tolerated by cells, causing high levels of cytotoxicity. Therefore, only results from transfections with N-terminal GFP are presented. A fusion protein of GFP linked to the N-terminus of the CREBBP bromodomain (amino acids 1,087 to 1,194) was not localised exclusively within the nucleus (data not shown) and was unsuitable for FRAP. Addition of the nuclear localisation sequence (NLS) from simian virus 40 (SV40) large T antigen between the GFP and the CREBBP bromodomain (Figure 4A, EX2) resulted in exclusively nuclear localisation, but the recovery time was rapid and the addition of SAHA resulted in only a moderate increase of FRAP recovery time. Furthermore, the assay was insensitive to inhibitor compounds (data not shown), possibly due to steric hindrance by the adjacent GFP tag. A larger construct spanning from the ends of the flanking domains (amino acids 868 to 1,341) and including a NLS (Figure 4A, EX3) also resulted in a very small assay window upon addition of SAHA (data not shown). To improve this assay window, we hypothesised that tandem repeats of the CREBBP bromodomain, like those seen in BET family members, might increase the apparent affinity of the chimeric CREBBP bromodomain protein for chromatin. An expression construct with N-terminal GFP and a NLS followed by three tandem repeats of the same amino acid sequence used in EX3 (Figure 4A, EX4) produced rapid recovery in FRAP with a t½ of 1.1 ± 0.1 seconds in wild-type control cells. Further reduction in recovery times was not seen when all three tandem repeats harboured the mutation corresponding to N1168F in full-length CREBBP (*P* > 0.05), but a substantial increase in the wild type was seen with the addition of SAHA (2.5 ± 0.1 seconds; *P* < 0.05). This increase was abolished in the bromodomain mutants (*P* < 0.05) and by addition of the inhibitor I-CBP112 at 1 μM [50] (*P* < 0.05), demonstrating that the tandem repeat construct provided both a good assay window and sensitivity to inhibitors.

**Figure 5:**
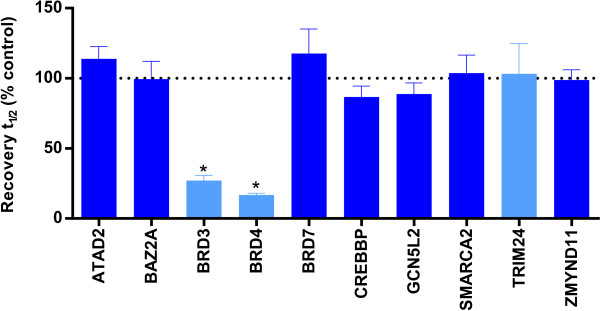
**Selectivity of fluorescence recovery after photobleaching assays.** Half-times of fluorescence recovery (t½) expressed as a percentage of the relevant wild-type control cells without inhibitor. Cells were transfected with the indicated bromodomain target and treated with 1 μM PFI-1. Light bars depict assays without suberoylanilide hydroxamic acid (SAHA) addition, and dark bars depict assays with SAHA addition (2.5 μM). The dotted line demarks the point equivalent to 100% of the relevant wild-type control cells without inhibitor. Error bars depict the standard error of the mean. Only bars marked with an asterisk indicate a significant difference from controls (*P* < 0.05).

To ascertain that selective inhibitors tested in FRAP experiments do not cross-react with other bromodomains in the cellular environment, the BET inhibitor PFI-1 was profiled across 10 bromodomain targets in FRAP assays at 1 μM. Significant reductions (*P* < 0.05) in FRAP recovery times relative to wild-type controls were observed only for the BET family members BRD3 and BRD4 (Figure 5). This finding is consistent with reported *in vitro* selectivity [18], demonstrating that the FRAP assays exhibit good selectivity for small-molecule inhibitors of bromodomains in intact cells.

Of the 42 bromodomain-containing proteins, subcellular localisation information is available for 29 of them in the Human Protein Atlas [40]. All but two of these proteins are found in the nucleus, although many, including BRD3, BRD4, TRIM24 and particularly BRD1, exhibit a granular, or even speckled, distribution. However, we have shown here that viable FRAP assays are still possible with these targets. BAZ1A and BRPF1, both of which are highly expressed in the testes, where they localise to the nucleus [51,52], appear to be largely excluded from the nuclei of cell lines, including U2OS cells [40], precluding FRAP assays with full-length proteins in common cell lines. These targets may be candidates for assays utilising bromodomain tandem repeats with the addition of a NLS, although the biological relevance of forcing these isolated bromodomains into the nucleus is questionable.

## Conclusions

Many bromodomains have identified roles in human disease, including cancer, inflammation and neurological disease, while the function and disease involvement of others remains unclear. This makes bromodomains attractive targets for chemical probe and novel therapeutic development. A key step in the drug discovery process is demonstration of cell permeability and *in vivo* efficacy. The 11 representative FRAP assays for bromodomain-containing proteins described here are distributed across the phylogenetic tree (Figure 1B) and demonstrate that FRAP cell-based assays are broadly applicable across this class of proteins, indicating that the method will be suitable for many other family members. Generation of bromodomain mutant controls can establish assay efficacy even in advance of inhibitor development. In situations where the binding contribution of the bromodomain is masked by other, stronger domains, the use of artificial tandem repeat constructs allows viable assays to be developed. These assays directly interrogate bromodomain binding to chromatin, not a downstream surrogate marker, while the short incubation time allows for measurement of bromodomain displacement only and not secondary effects due to subsequent inhibitor-induced changes in gene expression. Importantly, these assays can be implemented without the need for detailed knowledge of the function of the bromodomain-containing protein, which is lacking for many members of this family. Thus, FRAP assays offer a potentially universal method for generating cell-based assays for bromodomain-containing proteins that allow compounds to be tested for cell permeability and on-target efficacy. The method is also likely to be applicable to proteins containing other classes of epigenetic reader domains, including Tudor, PHD, chromo and MBT repeat domains.

## Methods

### Plasmids

The full-length cDNA clone for human *BRD4* [GenBank:NM_058243.2] was a gift from James Bradner (Department of Medical Oncology, Dana-Farber Cancer Institute, Boston, MA, USA). Full-length cDNAs were obtained as I.M.A.G.E. clones from Source BioScience Life Sciences (Nottingham, UK) for *ATAD2* [IMAGE:8322708] [GenBank:BC113656], *BAZ2A* [IMAGE:100015975] [GenBank:BC152739], *BRD1* [IMAGE: 100000034] [GenBank:CU013256], *BRD3* [IMAGE:4015879] [GenBank:BC031536], *BRD7* [IMAGE:100066308] GenBank:BC166008], *SMARCA2* [IMAGE:100061549] [GenBank:BC156185], *TRIM24* [IMAGE:5698079] [GenBank:BC056959] and *ZMYND11* [IMAGE:100003947] [GenBank:DQ891317]. Full-length cDNA for *CREBBP* [pFIKB0067] [GenBank:AB527452] was obtained from the Kazusa ORFeome Project (Kazusa DNA Research Institute, Kisarazu, Japan) [53]. Human clones were preferentially sourced (*ATAD2*, *BAZ2A*, *BRD4*, *CREBBP*, *SMARCA2* and *ZMYND11*), but where this was not possible, mouse clones were used (*BRD3*, *BRD7* and *TRIM24*). Where mouse clones were used, the amino acid sequences were >97% identical in the bromodomain region in all cases (see Additional file 2: Figure S2).

I.M.A.G.E. clones for *BAZ2A*, *BRD1*, *BRD7* and *SMARCA2* were in the pDONR223 backbone [54]. The I.M.A.G.E. clone for *ZMYND11* was in the pDONR221 backbone (Life Technologies, Carlsbad, CA, USA). For the remaining targets, each full-length gene was PCR-amplified (AccuPrime Pfx DNA Polymerase; Life Technologies) from the start codon to the stop codon with primers that included the appropriate attB sequences. PCR products were cloned with Gateway BP Clonase II enzyme mix (‘BP-cloned’ henceforth; Life Technologies) into either pDONR221 or pDONR223 to create entry clones (see Additional file 3: Table S1 for details of primers and DONR vectors). The gene of interest was then shuttled into either pcDNA5/FRT/TO-eGFP-DEST [55] (*ATAD2*, *BAZ2A*, *BRD1*, *BRD7*, *CREBBP*, *SMARCA2*, *TRIM24* and *ZMYND11*) or pcDNA6.2/N-EmGFP-DEST (*BRD3*, *BRD4* and *GCN5L2*) by cloning with the Gateway LR Clonase II Plus enzyme mix (‘LR-cloned’ henceforth; Life Technologies) to create chimeric GFP expression clones (see Additional file 4: Table S2).

Corresponding bromodomain mutant expression clones were generated using the megaprimer PCR method [56], taking advantage of either flanking restriction enzyme sites or att sites in the template. The exact position of the mutated residue is shown in Supplementary Figure 1. For cloning using flanking restriction enzyme sites, a first round of PCR using a primer outside the flanking region and a mutagenic primer with at least 10 base pairs (bp) of matching sequence on either side of the desired mutation was used to generate a megaprimer. A second round of PCR using the megaprimer and a primer beyond the opposite restriction site was used to generate a product flanked by both restriction sites and carrying the desired mutation. Both the PCR product and the original expression clone were digested with the appropriate restriction enzymes (New England Biolabs, Ipswich, MA, USA) and gel-purified. The expression clone was further treated with Antarctic phosphatase (New England Biolabs), before ligation with the PCR product by T4 DNA ligase (New England Biolabs) to create mutant chimeric GFP expression clones. I.M.A.G.E. clones for *BAZ2A* and *SMARCA2* lacked stop codons. Therefore, the derived expression clones were subjected to a prior round of mutagenesis to introduce stop codons. Details of templates, primers, restriction enzymes and resulting expression clones are summarised in Additional file 5: Table S3. For cloning using flanking att sites, a first round of PCR using a primer outside the flanking region and a mutagenic primer with at least 10 bp of matching sequence on either side of the desired mutation was used to generate a megaprimer. The decision whether the mutagenic primer was the sense or antisense primer was determined by the position of the mutation site so as to generate the smaller of the two possible megaprimers. A second round of PCR using the megaprimer and a primer beyond the second att site was used to generate a product flanked by both att sites and carrying the desired mutation. PCR products generated from pENTR templates were LR-cloned directly into pcDNA6.2/N-EmGFP-DEST to create chimeric GFP expression clones. PCR products generated from pDEST templates were BP-cloned into pDONR221, before subsequent LR cloning into pcDNA6.2/N-EmGFP-DEST, to create mutant chimeric GFP expression clones. Details of the templates, primers and cloning vectors used are summarised in Additional file 6: Table S4.

A *CREBBP* multimerised bromodomain construct was made using the MultiSite Gateway System [57] (Life Technologies). Three Gateway entry clones were created for both the wild-type and bromodomain mutant *CREBBP* that encompassed the region corresponding to RefSeq amino acids 868 to 1,341. The first entry clone also harboured the NLS from the SV40 large T antigen and was made by two rounds of PCR, the first using a sense primer incorporating the NLS and wild-type or mutant pcDNA5/FRT/TO-eGFP-DEST/CREBBP described above as the template and the second to add the appropriate attB sites to the PCR product, followed by BP cloning into the pDONR221 P1-P4 vector. The second and third entry clones did not carry NLS and were made directly by PCR amplification of wild-type or mutant pcDNA5/FRT/TO-eGFP-DEST/CREBBP with primers incorporating the appropriate attB sequences, followed by BP cloning into corresponding pDONR221 vectors. See Additional file 7: Table S5 for details of the primers and DONR vectors. The three entry clones for either the wild-type or mutant *CREBBP* bromodomain were combined by LR cloning into the pcDNA6.2/N-EmGFP-DEST vector to create an expression clone for three tandem repeats of the bromodomain fused to an N-terminal GFP (see Additional file 8: Table S6).

All constructs described are available upon request.

### Fluorescence recovery after photobleaching

FRAP studies were performed using a protocol modified from previous studies [17,18]. In brief, U2OS cells were reverse-transfected (Lipofectamine 2000 transfection reagent; Life Technologies) with mammalian overexpression constructs encoding bromodomain-containing proteins fused to GFP (wild type or mutants). Medium was replaced 6 hours after transfection with or without 2.5 μM SAHA, and 1 μM inhibitor was added 1 hour before imaging, which was carried out 24 hours after transfection. The FRAP and imaging system consisted of a Zeiss LSM 710 scan head (Zeiss GmbH, Jena, Germany) coupled to an inverted Zeiss Axio Observer.Z1 microscope equipped with a high numerical aperture (NA 1.3) 40× oil-immersion objective (Zeiss GmbH) and a heated chamber set at 37°C. Bleaching and GFP fluorescence imaging were carried out with an argon ion laser (488 nm) and a photomultiplier tube detector set to detect fluorescence between 500 and 550 nm. Cells with very high levels of GFP expression (saturation at gain settings >650) often displayed aberrant morphology and/or might be expected to have large pools of free GFP-tagged protein due to saturation of potential binding sites and were, therefore, excluded from selection for bleaching. Cells with very low levels of GFP expression requiring high gain settings (>850) resulted in noisy images and were particularly susceptible to photobleaching during imaging and were, therefore, also excluded from selection for bleaching. Thus, cells with nuclei just below saturation within the gain range of 650 to 850 were chosen for bleaching. A circular region of a GFP-positive nucleus was selected, and, after five prescans, the region was bleached. The size of the bleach area was altered for different bromodomain-containing proteins between 2.5 μm^2^ and 17.6 μm^2^ to keep t½ within a practical range of 1 to 30 seconds. A time-lapse series was then taken to record GFP recovery. During the time-lapse series, images were acquired with a frame size of 512 pixels × 512 pixels with line-stepping of 2, bidirectional scanning and a zoom factor of 6, which allowed for a time interval time of approximately 0.25 seconds. To decrease the level of photobleaching during acquisition, the laser was attenuated to just 1% of power used for bleaching, but with the pinhole diameter set to 1.39 airy units to improve detection of fluorescence.

The average intensity at each imaging time point was measured for three regions: the bleached region (*F*(*t*)_ROI_), the total cell nucleus (*F*(*t*)_total_) and a random region outside the cell for background subtraction (*F*(*t*)_BG_). The image data sets were exported from ZEN 2010 microscope control software (Zeiss GmbH) as text (.txt) files. The text files were batch-imported into OriginPro 9.1 software (OriginLab, Northampton, MA, USA) using a custom LabTalk script that performed all subsequent analysis. The relative fluorescence signal in the bleached region of each cell was calculated for each time point (*t*) with correction for photobleaching during image acquisition made using the method of Phair *et al*. [58], shown in equation 1, where *F*(i) is the mean intensity of a region in the five prebleach scans.

(1) $$F{\left(t\right)}_{\mathrm{norm}}=\;\frac{F{\left(t\right)}_{\mathrm{ROI}}\;-F{\left(t\right)}_{\mathrm{BG}}}{F{\left(t\right)}_{\mathrm{total}}\;-F{\left(t\right)}_{\mathrm{BG}}}\;\times \;\frac{F{\left(i\right)}_{\mathrm{total}}\;-F{\left(i\right)}_{\mathrm{BG}}}{F{\left(i\right)}_{\mathrm{ROI}}-F{\left(i\right)}_{\mathrm{BG}}}$$

Double exponential association curves were fitted to the normalised data using the OriginPro function ExpAssoc, which has the formula shown in equation 2.

(2) $$y=y0+A1\left(1-e^{-x/t1}\right)+A2\left(1-e^{-x/t2}\right)$$

Using parameters returned by the curve-fitting, the fluorescence intensity corresponding to half-recovery was calculated using equation 3.

(3) $$y½=y0+\frac{A1+A2}{2}$$

An iterative bisection algorithm was then used to determine the value of *x* (that is, time for half recovery, or t_½_) in equation 2 when *y* = *y*½.

Because equation 1 normalises data to prebleach intensities, plateaus from curve-fitting should not be >1. However, focal drift during FRAP could shift plateaus substantially above 1, and cells which moved during FRAP or out of focus debris passing over the cell during imaging resulted in irregular recovery profiles and poor curve-fitting. Therefore, cells where curve-fitting gave plateaus >1.1 or adjusted *R*^2^ values <0.95 were removed from further analysis as imaging artefacts. Outliers within a treatment group were eliminated using the Grubbs test [59] before the calculation of the arithmetic mean of each treatment group. One-way ANOVA with Tukey–Kramer correction for multiple comparisons was used to detect significant differences (*P* < 0.05) between treatment groups.

## Abbreviations

FRAP: Fluorescence recovery after photobleaching; GFP: Green fluorescent protein; HAT: Histone acetyltransferase; HDAC: Histone deacetylase; NLS: Nuclear localisation signal; SAHA: Suberoylanilide hydroxamic acid; t½: Half-time for FRAP; wt: Wild type.

## Competing interests

The authors declare that they have no competing interests.

## Authors’ contributions

MP contributed to study design, made wild-type and mutant constructs, performed FRAP experiments, analysed data and wrote the manuscript. CR and CW performed FRAP experiments. CY optimised confocal microscopy for FRAP. JPL and ACG made wild-type constructs. CSD and NBB made mutant constructs. NBB contributed to study design. SK and SM contributed to study design and manuscript writing. All authors read and approved the final manuscript.

## Supplementary Material

Additional file 1: Figure S1 Suberoylanilide hydroxamic acid dose response. Effect of SAHA concentration on FRAP recovery time in U2OS cells transfected with plasmids encoding GFP chimerised to wild-type or mutant SMARCA2. *P < 0.05, significant difference from wt. Click here for file

Additional file 2: Figure S2 Alignment of bromodomain protein sequences. Where murine genes have been used for FRAP constructs, human equivalents are included in the alignment. Red arrow denotes position of mutagenesis. Click here for file

Additional file 3: Table S1 Primer and pDONR details for BP cloning. Click here for file

Additional file 4: Table S2 LR cloning of full-length GFP chimeric constructs. Click here for file

Additional file 5: Table S3 Primer details for mutagenesis and restriction enzymes used for cloning. Click here for file

Additional file 6: Table S4 Primer details for mutagenesis and pDEST used for LR cloning. Click here for file

Additional file 7: Table S5 Primer and pDONR details for generation of multimerised CREBBP bromodomain construct. Click here for file

Additional file 8: Table S6 Details of LR cloning of multimerised bromodomain constructs. Click here for file
