# Supplementary figures and images for: Assessing cellular efficacy of bromodomain inhibitors using fluorescence recovery after photobleaching
Source: Epigenetics Chromatin. 2014 Jul 13;7:14. doi: 10.1186/1756-8935-7-14 (PMC4115480; doi:10.1186/1756-8935-7-14)

**Additional File 1: Figure S1**

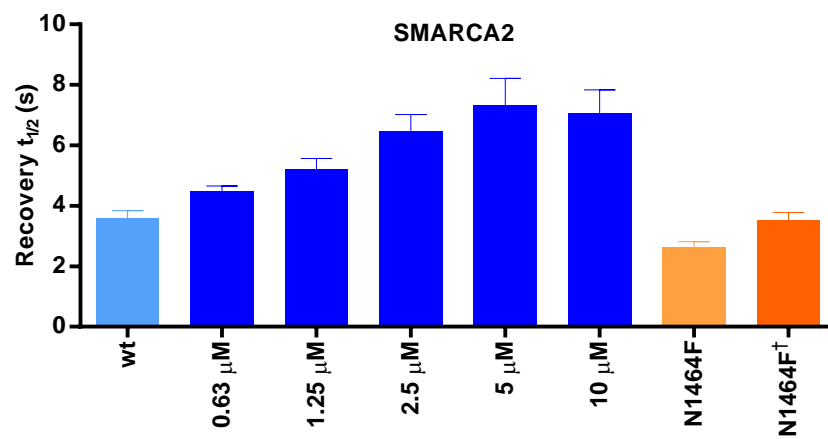

Supplement: Additional file 1: Figure S1 — Suberoylanilide hydroxamic acid dose response. Effect of SAHA concentration on FRAP recovery time in U2OS cells transfected with plasmids encoding GFP chimerised to wild-type or mutant SMARCA2. *P < 0.05, significant difference from wt. [file 1756-8935-7-14-S1.pdf]
