# Supplementary material for: Assessing cellular efficacy of bromodomain inhibitors using fluorescence recovery after photobleaching
Source: Epigenetics Chromatin. 2014 Jul 13;7:14. doi: 10.1186/1756-8935-7-14 (PMC4115480; doi:10.1186/1756-8935-7-14)
Supplement: Additional file 2: Figure S2 — Alignment of bromodomain protein sequences. Where murine genes have been used for FRAP constructs, human equivalents are included in the alignment. Red arrow denotes position of mutagenesis. [file 1756-8935-7-14-S2.pdf]

Additional File 2: Figure S2

```
                   (1) 1        10        20        30        40        50        60        70        80        90        100       110       122
ATAD2 bromo        (1) -------RELRIFLRNVTHRLAI--DKRFRVFTKPVDPDEVPDYVTVIKQPMDLSSVISKIDLHKYLT--VKDYLRDIDLICSNALEYNPDRDPGDRLIRHRACALRDTAYAIIKEELDEDF
GCN5L2 bromo       (1) ----QLYTTLKNLLAQIKS------HPSAWPFMEPVKKSEAPDYYEVIRFPIDLKTMTERLRSRYYVTR--KLFVADLQRVIANCREYNPP----DSEYCRCASALEKFFYFKLKE------
BRD1 bromo         (1) ----PLTVLLRSVLDQLQD------KDPARIFAQPVSLKEVPDYLDHIKHPMDFATMRKRLEAQGYKN--LHEFEEDFDLIIDNCMKYNAR----DTVFYRAAVRLRDQGGVVL--------
BRD7 bromo         (1) ----PLQEALNQLMRQLQR------KDPSAFFSFPVTDFIAPGYSMIIKHPMDFSTMKEKIKNNDYQS--IEELKDNFKLMCTNAMIYNKP----ETIYYKAAKKLLHSGMKIL--------
hBRD7 bromo        (1) ----PLQEALNQLMRQLQR------KDPSAFFSFPVTDFIAPGYSMIIKHPMDFSTMKEKIKNNDYQS--IEELKDNFKLMCTNAMIYNKP----ETIYYKAAKKLLHSGMKIL--------
mBRD7 bromo        (1) ----PLQEALNQLMRQLQR------KDPSAFFSFPVTDFIAPGYSMIIKHPMDFSTMKEKIKNNDYQS--IEELKDNFKLMCTNAMIYNKP----ETIYYKAAKKLLHSGMKIL--------
BAZ2A bromo        (1) ----DLTFCEIILMEMESH---D--AAWPFLEPVNPRLVSG--YRRIIKNPMDFSTMRERLLRGGYTS--SEEFAADALLVFDNCQTFNED----DSEVGKAGHIMRRFFESRW--------
hTRIM24 bromo      (1) LTPIDKRKCERLLLFLYCH--------EMSLAFQDPVPLTVPDYYKIIKNPMDLSTIKKRLQEDYSMYSKPEDFVADFRLIFQNCAEFNEP----DSEVANAGIKLENYFEELLKNLYPE--
mTRIM24 bromo      (1) LTPIDKRKCERLLLFLYCH--------EMSLAFQDPVPLTVPDYYKIIKNPMDLSTIKKRLQEDYCMYTKPEDFVADFRLIFQNCAEFNEP----DSEVANAGIKLESYFEELLKNLYPE--
BRD4 bromo 1       (1) RQTNQLQYLLRVVLKTLWK--HQ--FAWPFQQPVDAVKLNLPDYYKIIKTPMDMGTIKKRLENNYYWN--AQECIQDFNTMFTNCYIYNKP----GDDIVLMAEALEKLFLQKINEL-----
hBRD3 bromo 1      (1) RKTNQLQYMQNVVVKTLWK--HQ--FAWPFYQPVDAIKLNLPDYHKIIKNPMDMGTIKKRLENNYYWS--ASECMQDFNTMFTNCYIYNKP----TDDIVLMAQALEKIFLQKVAQM-----
mBRD3 bromo 1      (1) RKTNQLQYMQNVVVKTLWK--HQ--FAWPFYQPVDAIKLNLPDYHKIIKNPMDMGTIKKRLENNYYWS--ASECMQDFNTMFTNCYIYNKP----TDDIVLMAQALEKIFLQKVAQM-----
BRD4 bromo 2       (1) ----QLKCCSGILKEMFAKKHAA--YAWPFYKPVDVEALGLHDYCDIIKHPMDMSTIKSKLEAREYRD--AQEFGADVRLMFSNCYKYNPP----DHEVVAMARKLQDVFEMRF--------
hBRD3 bromo 2      (1) ----HLRYCDSILREMLSKKHAA--YAWPFYKPVDAEALELHDYHDIIKHPMDLSTVKRKMDGREYPD--AQGFAADVRLMFSNCYKYNPP----DHEVVAMARKLQDVFEMRF--------
mBRD3 bromo 2      (1) ----HLRHCDSILREMLSKKHAA--YAWPFYKPVDAEALELHDYHDIIKHPMDLSTVKRKMDSREYPD--AQGFAADIRLMFSNCYKYNPP----DHEVVAMARKLQDVFEMRF--------
CREBBP bromo       (1) --PEELRQALMPTLEALYR-QDP--ESLPFRQPVDPQLLGIPDYFDIVKNPMDLSTIKRKLDTGQYQEP--WQYVDDVWLMFNNAWLYNRK----TSRVYKFCSKLAEVFEQEIDPVMQ---
SMARCA2 bromo      (1) ----KLTKQMNAIIDTVINYKDSSGRQLSEVFIQLPSRKELPEYYELIRKPVDFKKIKERIRNHKYRS--LGDLEKDVMLLCHNAQTFNLE----GSQIYEDSIVLQSVFKSARQKI-----
ZMYND11 bromo      (1) -----MGTYLRFIVSRMKER--------AIDLNKKGKDNKHPMYRRLVHSAVDVPTIQEKVNEGKYRS--YEEFKADAQLLLHNTVIFYGDSEQADIARMLYKDTCHELDELQLC-------
Consensus          (1)      L   L ILL  L K          F    D   L LPDYY IIK PMDLSTIKKKLE   Y S    EF  DF LMFTNC  YN P    DSEV  A KL  VF   L
```
