# Supplementary material for: Assessing cellular efficacy of bromodomain inhibitors using fluorescence recovery after photobleaching
Source: Epigenetics Chromatin. 2014 Jul 13;7:14. doi: 10.1186/1756-8935-7-14 (PMC4115480; doi:10.1186/1756-8935-7-14)
Supplement: Additional file 3: Table S1 — Primer and pDONR details for BP cloning. [file 1756-8935-7-14-S3.pdf]

Additional File 3: Table S1

Gene	Template	Primer	Sequence	pDONR	Entry Clone
ATAD2	I.M.A.G.E. 8322708	5’	GGGGACAAGTTTGTACAAAAAAGCAGGCTTAATGGTGGTTCTCCGCAGC	pDONR223	pENTR223/ ATAD2
		3’	GGGGACCACTTTGTACAAGAAAGCTGGGTATCATCTGGAACAACTGAA		
BRD3	I.M.A.G.E. 4015879	5’	GGGGACAAGTTTGTACAAAAAAGCAGGCTTAATGGGCAAAAAGCACAAGAAGCA	pDONR223	pENTR223/ BRD3
		3’	GGGGACCACTTTGTACAAGAAAGCTGGGTATTAGTTCTTGGCAGGAGCTG		
BRD4	NM_058243.2	5’	GGGGACAAGTTTGTACAAAAAAGCAGGCTTAATGTCTGCGGAGAGCGGC	pDONR221	pENTR221/ BRD4
		3’	GGGGACCACTTTGTACAAGAAAGCTGGGTATCAGAAAAGATTTTCTTC		
CREBBP	pFIKB0067	5’	GGGGACAAGTTTGTACAAAAAAGCAGGCTTAATGGCTGAGAACTTGCTG	pDONR223	pENTR223/ CREBBP
		3’	GGGGACCACTTTGTACAAGAAAGCTGGGTATTAAACCAAGCCCTCCACAAACT		
GCN5L2	I.M.A.G.E. 6137532	5’	GGGGACAAGTTTGTACAAAAAAGCAGGCTTAATGGCGGAACCTTCCCAG	pDONR223	pENTR223/ GCN5L2
		3’	GGGGACCACTTTGTACAAGAAAGCTGGGTACTACTTGTCAATGAGGCC		
TRIM24	I.M.A.G.E. 5698079	5’	GGGGACAAGTTTGTACAAAAAAGCAGGCTTAATGGAGGTGGCTGTGGAG	pDONR223	pENTR223/ TRIM24
		3’	GGGGACCACTTTGTACAAGAAAGCTGGGTATTACTTAAGCAGCTGGCGATCCTC		
