# Supplementary material for: Assessing cellular efficacy of bromodomain inhibitors using fluorescence recovery after photobleaching
Source: Epigenetics Chromatin. 2014 Jul 13;7:14. doi: 10.1186/1756-8935-7-14 (PMC4115480; doi:10.1186/1756-8935-7-14)
Supplement: Additional file 4: Table S2 — LR cloning of full-length GFP chimeric constructs. [file 1756-8935-7-14-S4.pdf]

Additional File 4: Table S2

Gene	Entry Clone	pDEST	Expression Clone
ATAD2	pENTR223/ATAD2	pcDNA5/FRT/TO-eGFP-DEST	pcDNA5/FRT/TO-eGFP-DEST/ATAD2
BAZ2A	I.M.A.G.E. 100015975	pcDNA5/FRT/TO-eGFP-DEST	pcDNA5/FRT/TO-eGFP-DEST/BAZ2A
BRD1	I.M.A.G.E. 100000034	pcDNA5/FRT/TO-eGFP-DEST	pcDNA5/FRT/TO-eGFP-DEST/BRD1
BRD3	pENTR223/BRD3	pcDNA6.2/N-EmGFP-DEST	pcDNA6.2/N-EmGFP-DEST/BRD3
BRD4	pENTR221/BRD4	pcDNA6.2/N-EmGFP-DEST	pcDNA6.2/N-EmGFP-DEST/BRD4
BRD7	I.M.A.G.E. 10006630	pcDNA6.2/N-EmGFP-DEST	pcDNA5/FRT/TO-eGFP-DEST /BRD7
CREBBP	pENTR223/CREBBP	pcDNA5/FRT/TO-eGFP-DEST	pcDNA5/FRT/TO-eGFP-DEST/CREBBP
GCN5L2	pENTR223/GCN5L2	pcDNA6.2/N-EmGFP-DEST	pcDNA6.2/N-EmGFP-DEST/GCN5L2
SMARCA2	I.M.A.G.E. 100061549	pcDNA5/FRT/TO-eGFP-DEST	pcDNA5/FRT/TO-eGFP-DEST/SMARCA2
TRIM24	pENTR223/TRIM24	pcDNA5/FRT/TO-eGFP-DEST	pcDNA5/FRT/TO-eGFP-DEST/TRIM24
ZMYND11	I.M.A.G.E. 100003947	pcDNA6.2/N-EmGFP-DEST	pcDNA5/FRT/TO-eGFP-DEST /ZMYND11
