# Supplementary material for: Assessing cellular efficacy of bromodomain inhibitors using fluorescence recovery after photobleaching
Source: Epigenetics Chromatin. 2014 Jul 13;7:14. doi: 10.1186/1756-8935-7-14 (PMC4115480; doi:10.1186/1756-8935-7-14)
Supplement: Additional file 5: Table S3 — Primer details for mutagenesis and restriction enzymes used for cloning. [file 1756-8935-7-14-S5.pdf]

Additional File 5: Table S3

Gene	Template	Primer	Sequence	Digest	Product
ATAD2	pcDNA5/FRT/TO-eGFP-DEST/ ATAD2	1st 5’	GAAGAGGTGCAAGAATTGTTTATCC	BlpI/ RsrII	pcDNA5/FRT/TO-eGFP-DEST/ ATAD2 N1064F
		1st 3’	GGATCTCTATCTGGGAAGTATTCTAAGG		
		2nd 5’	megaprimer (1st PCR)		
		2nd 3’	CACGCCATGTAGTGTATTGACCG		
BAZ2A	pcDNA5/FRT/TO-eGFP-DEST/ BAZ2A (no stop)	1st 5’	CAGGCCAATCTGTGAGGCCTCATGGGC	EcoRI/ SphI	pcDNA5/FRT/TO-eGFP-DEST/ BAZ2A (stop)
		1st 3’	CCTCAGAAGCCATAGAGCC		
		2nd 5’	CTGTTTGGCTCAGCAGGTGG		
		2nd 3’	megaprimer (1st PCR)		
BAZ2A	pcDNA5/FRT/TO-eGFP-DEST/ BAZ2A (stop)	1st 5’	CCAGACTTTCTTCGAGGATGATTC	EcoRI/ SphI	pcDNA5/FRT/TO-eGFP-DEST/ BAZ2A N1873F
		1st 3’	GCTGGTTCTTTCCGCCTCAGAAGC		
		2nd 5’	GCTGCCACATTTACTGCCATCGTCC		
		2nd 3’	megaprimer (1st PCR)		
BRD1	pcDNA5/FRT/TO-eGFP-DEST/ BRD1	1st 5’	CATGAAGTACGCAGCCAGGGACA	BamHI/ BstBI	pcDNA5/FRT/TO-eGFP-DEST/ BRD1 N642A
		1st 3’	CTCCGTCCTCTTCGAAGCCT		
		2nd 5’	TGTGCCCTGTGGATCCCAGA		
		2nd 3’	megaprimer (1st PCR)		
CREBBP	pcDNA5/FRT/TO-eGFP-DEST/ CREBBP	1st 5’	GCCTGGCTCTATTTCCGCAAGACATCCCG	NsiI/ BstEII	pcDNA5/FRT/TO-eGFP-DEST/ CREBBP N1168F
		1st 3’	GCAGAACGCAAATCTGATGC		
		2nd 5’	CCTACAGTCCAGGCAGCAGC		
		2nd 3’	megaprimer (1st PCR)		
SMARCA2	pcDNA5/FRT/TO-eGFP-DEST/ SMARCA2 (no stop)	1st 5’	GGACGGATGATGAGTGAGGCCTCATGGG	SbfI/ XhoI	pcDNA5/FRT/TO-eGFP-DEST/ SMARCA2 (stop)
		1st 3’	GCAACTAGAAGGCACAGTCG		
		2nd 5’	GTCTTCAAGCCACGAGCGGAG		
		2nd 3’	megaprimer (1st PCR)		
SMARCA2	pcDNA5/FRT/TO-eGFP-DEST/ SMARCA2 (stop)	1st 5’	CTCAGACGTTCTTCCTGGAGGGATCCC	SbfI/ XhoI	pcDNA5/FRT/TO-eGFP-DEST/ SMARCA2 N1464F
		1st 3’	GCAACTAGAAGGCACAGTCG		
		2nd 5’	GTCTTCAAGCCACGAGCGGAG		
		2nd 3’	megaprimer (1st PCR)		

TRIM24	pcDNA5/FRT/TO-eGFP-DEST/ TRIM24	1st 5’<br>1st 3’<br>2nd 5’<br>2nd 3’	GTGCTGAATTCTTCGAGCCTGATTCTG<br>GCACAGTCGAGGCTGATCAGC<br>CCATCTTACTTGTCATGTGC<br>megaprimer (1st PCR)	XhoI/ XcmI	pcDNA5/FRT/TO-eGFP-DEST/ TRIM24 N981F
