# Supplementary material for: Assessing cellular efficacy of bromodomain inhibitors using fluorescence recovery after photobleaching
Source: Epigenetics Chromatin. 2014 Jul 13;7:14. doi: 10.1186/1756-8935-7-14 (PMC4115480; doi:10.1186/1756-8935-7-14)
Supplement: Additional file 6: Table S4 — Primer details for mutagenesis and pDEST used for LR cloning. [file 1756-8935-7-14-S6.pdf]

Additional File 6: Table S4

Gene	Template	Primer	Sequence	pDONR/ pDEST	Product
BRD3	pENTR223/<br>BRD3	1st 5'<br>1st 3'<br>2nd 5'<br>2nd 3'	TGTAAAACGACGGCCAGTCT<br>CTGTGGGCTTGAAATAAATATAAC<br>megaprimer (1st PCR)<br>CAGGAAACAGCTATGACCATG	pcDNA6.2/<br>N-EmGFP-DEST	pcDNA6.2/N-EmGFP-<br>DEST/BRD3 N115F
BRD3	pENTR223/<br>BRD3	1st 5'<br>1st 3'<br>2nd 5'<br>2nd 3'	TGTAAAACGACGGCCAGTCT<br>GGTCTGGAGGGAAGTACTTATAAC<br>megaprimer (1st PCR)<br>CAGGAAACAGCTATGACCATG	pcDNA6.2/<br>N-EmGFP-DEST	pcDNA6.2/N-EmGFP-<br>DEST/BRD3 N390F
BRD3	PCR product used to create BRD3 N390F mutant (immediately above)	1st 5'<br>1st 3'<br>2nd 5'<br>2nd 3'	TGTAAAACGACGGCCAGTCT<br>CTGTGGGCTTGAAATAAATATAAC<br>megaprimer (1st PCR)<br>CAGGAAACAGCTATGACCATG	pcDNA6.2/<br>N-EmGFP-DEST	pcDNA6.2/N-EmGFP-<br>DEST/BRD3 N115F-N390F
BRD4	pENTR221/<br>BRD4	1st 5'<br>1st 3'<br>2nd 5'<br>2nd 3'	TGTAAAACGACGGCCAGTCT<br>CTCCAGGCTTGAAGTAGATGTAA<br>megaprimer (1st PCR)<br>CAGGAAACAGCTATGACCATG	pcDNA6.2/<br>N-EmGFP-DEST	pcDNA6.2/N-EmGFP-<br>DEST/BRD4 N140F
BRD4	pENTR221/<br>BRD4	1st 5'<br>1st 3'<br>2nd 5'<br>2nd 3'	TGTAAAACGACGGCCAGTCT<br>GGTCAGGAGGGAAGTACTTATAG<br>megaprimer (1st PCR)<br>CAGGAAACAGCTATGACCATG	pcDNA6.2/<br>N-EmGFP-DEST	pcDNA6.2/N-EmGFP-<br>DEST/BRD4 N433F
BRD4	PCR product used to create BRD4 N433F mutant (immediately above)	1st 5'<br>1st 3'<br>2nd 5'<br>2nd 3'	TGTAAAACGACGGCCAGTCT<br>CTCCAGGCTTGAAGTAGATGTAA<br>megaprimer (1st PCR)<br>CAGGAAACAGCTATGACCATG	pcDNA6.2/<br>N-EmGFP-DEST	pcDNA6.2/N-EmGFP-<br>DEST/BRD4 N140F-N433F
BRD7	pcDNA5/FRT/TO-<br>eGFP-DEST/ BRD7	1st 5'<br>1st 3'<br>2nd 5'<br>2nd 3'	GGCGCGCCCACAAGTTTGTA<br>TCTCTGGCTTGAAGTAAATCATTGC<br>megaprimer (1st PCR)<br>AGGCACAGTCGAGGCTGATCA	pDONR221	pENTR221/BRD7 N211F

GCN5L2	pENTR223/<br>GCN5L2	1st 5’<br>1st 3’<br>2nd 5’<br>2nd 3’	TCGCGAGTACTTCCCCCCGGACA<br>TATCGCGAGCCCATTTATAC<br>TGTAAAACGACGGCCAGTCT<br>megaprimer (1st PCR)	pcDNA6.2/<br>N-EmGFP-DEST	pcDNA6.2/N-EmGFP-DEST/GCN5L2 N808F
ZMYND11	pcDNA5/FRT/TO-eGFP-DEST/ZMYND11	1st 5’<br>1st 3’<br>2nd 5’<br>2nd 3’	GGCGCGCCCACAAGTTTGTA<br>CACTGTCTCCGAAGAAAATCACGG<br>megaprimer (1st PCR)<br>AGGCACAGTCGAGGCTGATCA	pDONR221	pENTR221/ZMYND11 Y191A
