# Supplementary material for: Assessing cellular efficacy of bromodomain inhibitors using fluorescence recovery after photobleaching
Source: Epigenetics Chromatin. 2014 Jul 13;7:14. doi: 10.1186/1756-8935-7-14 (PMC4115480; doi:10.1186/1756-8935-7-14)
Supplement: Additional file 7: Table S5 — Primer and pDONR details for generation of multimerised CREBBP bromodomain construct. [file 1756-8935-7-14-S7.pdf]

Additional File 7: Table S5

Template	Primer	Sequence	pDONR	Entry Clone
pcDNA5/FRT/TO-eGFP-DEST/CREBBP	5’<br>3’	ATGCCCAAGAAGAAGAGGAAAGTCTCTCTCCAGCACACGACA<br>TCGGTCTTCCAAGTGGTTTCCCAGTCTT	N/A	N/A
PCR product (immediately above)	5’<br>3’	GGGGACAAGTTTGTACAAAAAAGCAGGCTTAATGCCCAAGAAGAAGAGG<br>GGGGACAACTTTGTATAGAAAAGTTGGGTGTCGGTCTTCCAAGTGGTT	pDONR221 P1-P4	pENTR221 P1-P4/NLS/ CREBBP aa868-1341
pcDNA5/FRT/TO-eGFP-DEST/CREBBP	5’<br>3’	GGGGACAACTTTTCTATACAAAGTTGCTCTCCAGCACACGACACCA<br>GGGGACAACTTTATTATACAAAGTTGTTCGGTCTTCCAAGTGGTT	pDONR221 P4r-P3r	pENTR221 P4r-P3r/ CREBBP aa868-1341
pcDNA5/FRT/TO-eGFP-DEST/CREBBP	5’<br>3’	GGGGACAACTTTGTATAATAAAGTTGCTCTCCAGCACACGACACCA<br>GGGGACCACTTTGTACAAGAAAGCTGGGTACTATCGGTCTTCCAAGTGGTT	pDONR221 P3-P2	pENTR221 P3-P2/ CREBBP aa868-1341
pcDNA5/FRT/TO-eGFP-DEST/ CREBBP N1168	5’<br>3’	ATGCCCAAGAAGAAGAGGAAAGTCTCTCTCCAGCACACGACA<br>TCGGTCTTCCAAGTGGTTTCCCAGTCTT	N/A	N/A
PCR product (immediately above)	5’<br>3’	GGGGACAAGTTTGTACAAAAAAGCAGGCTTAATGCCCAAGAAGAAGAGG<br>GGGGACAACTTTGTATAGAAAAGTTGGGTGTCGGTCTTCCAAGTGGTT	pDONR221 P1-P4	pENTR221 P1-P4/NLS/ CREBBP aa868-1341 N1168F
pcDNA5/FRT/TO-eGFP-DEST/ CREBBP N1168	5’<br>3’	GGGGACAACTTTTCTATACAAAGTTGCTCTCCAGCACACGACACCA<br>GGGGACAACTTTATTATACAAAGTTGTTCGGTCTTCCAAGTGGTT	pDONR221 P4r-P3r	pENTR221 P4r-P3r/CREBBP aa868-1341
pcDNA5/FRT/TO-eGFP-DEST/ CREBBP N1168	5’<br>3’	GGGGACAACTTTGTATAATAAAGTTGCTCTCCAGCACACGACACCA<br>GGGGACCACTTTGTACAAGAAAGCTGGGTACTATCGGTCTTCCAAGTGGTT	pDONR221 P3-P2	pENTR221 P3-P2/ CREBBP aa868-1341
