# Supplementary material for: Assessing cellular efficacy of bromodomain inhibitors using fluorescence recovery after photobleaching
Source: Epigenetics Chromatin. 2014 Jul 13;7:14. doi: 10.1186/1756-8935-7-14 (PMC4115480; doi:10.1186/1756-8935-7-14)
Supplement: Additional file 8: Table S6 — Details of LR cloning of multimerised bromodomain constructs. [file 1756-8935-7-14-S8.pdf]

Additional File 8: Table S6

pENTR clone 1	pENTR clone 2	pENTR clone 3	pDEST	Expression Clone
pENTR221 P1-P4/NLS/ CREBBP aa868-1341	pENTR221 P4r-P3r/ CREBBP aa868-1341	pENTR221 P3-P2/ CREBBP aa868-1341	pcDNA6.2/N-EmGFP-DEST	pcDNA6.2/N-EmGFP-DEST/3x CREBBP aa868-1341
pENTR221 P1-P4/NLS/ CREBBP aa868-1341 N1168F	pENTR221 P4r-P3r/CREBBP aa868-1341	pENTR221 P3-P2/ CREBBP aa868-1341	pcDNA6.2/N-EmGFP-DEST	pcDNA6.2/N-EmGFP-DEST/3x CREBBP aa868-1341 N1168F
